# Eco‐Friendly and High Performance Supercapacitors for Elevated Temperature Applications Using Recycled Tea Leaves

**DOI:** 10.1002/gch2.201700063

**Published:** 2017-10-09

**Authors:** Sanket Bhoyate, Charith K. Ranaweera, Chunyang Zhang, Tucker Morey, Megan Hyatt, Pawan K. Kahol, Madhav Ghimire, Sanjay R. Mishra, Ram K. Gupta

**Affiliations:** ^1^ Department of Chemistry Pittsburg State University Pittsburg KS 66762 USA; ^2^ Labette County High School Altamont KS 67330 USA; ^3^ Department of Physics Pittsburg State University Pittsburg KS 66762 USA; ^4^ Department of Physics and Materials Science The University of Memphis Memphis TN 38152 USA; ^5^ Kansas Polymer Research Center Pittsburg State University Pittsburg KS 66762 USA

**Keywords:** activated carbon, biowaste, energy storage, supercapacitor, tea leaves

## Abstract

Used tea leaves are utilized for preparation of carbon with high surface area and electrochemical properties. Surface area and pore size of tea leaves derived carbon are controlled by varying the amount of KOH as activating agent. The maximum surface area of 2532 m^2^ g^−1^ is observed, which is much higher than unactivated tea leaves (3.6 m^2^ g^−1^). It is observed that the size of the electrolyte ions has a profound effect on the energy storage capacity. The maximum specific capacitance of 292 F g^−1^ is observed in 3 m KOH electrolyte with outstanding cyclic stability, while the lowest specific capacitance of 246 F g^−1^ is obtained in 3 m LiOH electrolyte at 2 mV s^−1^. The tea leaves derived electrode shows almost 100% capacitance retention up to 5000 cycles of study. The symmetrical supercapacitor device shows a maximum specific capacitance of 0.64 F cm^−2^ at 1 mA cm^−2^ and about 95% of specific capacitance is retained after increasing current density to 12 mA cm^−2^, confirming the high rate stability of the device. An improvement over 35% in the charge storage capacity is seen when increasing device temperature from 10 to 80 °C. The study suggests that used tea leaves can be used for the fabrication of environment friendly high performance supercapacitor devices at a low cost.

## 1 Introduction

Portable or remote gadgets require continuous power supply creating a demand for local electrical energy production or storage. With increasing use of handhold electronic devices, desirable factors such as light weight, high performance, and consistent energy supply are required. Hence, an efficient local energy storage/production system plays a vital role for such applications.^1^ The supercapacitor is one of the efficient energy storage systems which can store energy via electric double layer and faradic reactions.^2^, ^3^ The electric double layer capacitor (EDLC) stores the charge over the active surface area of the material and shows very durable performance over long period.^3^, ^4^ Thus, creating carbon with a high surface area could be one of the ways to improve the performance of EDLC‐based supercapacitor devices. There are several publications documenting the utilization of waste‐derived carbon source such as seeds, seed shells, leaves, bagasse, waste paper, and tyre.^5^, ^6^, ^7^, ^8^, ^9^, ^10^, ^11^, ^12^ Waste tea is an additional exciting source for carbon.^13^ Most of the time, an activation step is required to achieve the high surface area to enhance the performance of the carbon. Activation of waste‐derived carbon can be done using various methods including chemical, steam, and CO_2_‐based activation.^14^, ^15^, ^16^, ^17^ For example, steam activated carbon from fir wood showed an activated surface area of 1131 m^2^ g^−1^ while, CO_2_ activated empty fruit bunches of palm showed a surface area of 1704 m^2^ g^−1^.^12^, ^13^ KOH activation is the most popular way to create high surface area and porosity.^18^ KOH activated carbon from rice bran showed a high surface area of 2475 m^2^ g^−1^.^15^, ^16^, ^19^

Several KOH‐based activated carbon derivations from various sources for supercapacitor applications have been reported. For example, Kalpana et al. reported an electric double layer capacitor made from KOH activated carbon using waste newspaper.^20^ Newspaper‐derived carbon showed a surface area of 416 m^2^ g^−1^ and specific capacitance of 180 F g^−1^. Ferrero et al. produced environmental friendly electrode materials from soybean residue with KOH activation resulting in a high surface area of 1950 m^2^ g^−1^ and exhibited a high specific capacitance of 260 F g^−1^ in 1 m H_2_SO_4_ electrolyte.^21^ Li et al. used sunflower seed shell for production of KOH activated nanoporous carbon and studied different activation schemes namely, impregnation‐activation process and carbonization activation process. Specific capacitance of 311 F g^−1^ in KOH electrolyte was observed for activated carbon with a specific surface area of 2509 m^2^ g^−1^.^6^ Carbon from biomass such as glucose was used to improve the performance of ferroferric oxide nanorods by providing carbon‐shell protection to the metal oxide nanorodes.^22^

Deriving surface area from micropores has been very effective in increasing the charge storage efficiency of the carbon.^6^ Low cost and inherently porous carbon materials are challenging sources to obtain from nature. Furthermore, the type of activating agent and characteristics of the pores in carbon and electrolytes used affect the capacitive performance.^5^, ^14^ Hence, apart from activation at different KOH ratios, it is also essential to understand the electrochemical behavior of activated carbon in different aqueous electrolytes. In this study, we report on structural and electrochemical properties of carbon prepared by calcination and activation of waste tea leaves. Activation was done by varying the tea leaves to KOH ratio (1:1, 1:2, and 1:3). The electrochemical study of activated bioporous tea leaves derived carbon was performed by using cyclic voltammetry (CV), galvanostatic charge–discharge (GCD), and electrochemical impedance spectroscopy (EIS). Stability tests of activated carbon from tea leaves showed stable performance for 5000 cycles and improved capacitive performance raising temperatures from 10 to 80 °C suggesting its potential to be used for high‐temperature applications. Further, the device showed high energy and power density of 0.089 mWh cm^−2^ (761 Wh kg^−1^) and 6 mW cm^−2^ (11.1 W kg^−1^), respectively, displaying its potential to be used for bio‐based future supercapacitor devices.

## 2 Result and Discussion

**Figure 1:**
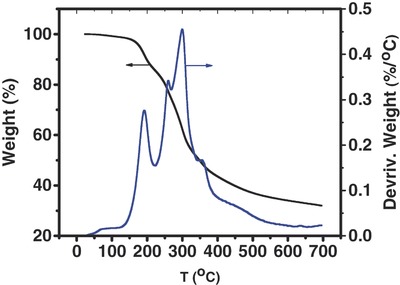
Thermogravimetric and its derivative curves for tea leaves.

Thermal behavior of tea leaves was studied using thermogravimetric analysis. Thermogravimetric (TG) and its derivative (DTG) curves for tea leaves are shown in **Figure** **1**. Decomposition peaks for different samples can be observed from the DTG curves of the tea leaves. The decomposition peaks cantered around 190, 260, 300, and 360 °C could be due to dehydration, decomposition of hemicellulose, and cellulose of the tea leaves.^23^, ^24^ A peak in temperature range of 50–200 °C could be due to dehydration of the tea leaves. With a further increase in temperature, three peaks around 260, 300, and 360 °C were observed which could be due to pyrolysis of hemicellulose, cellulose, and lignin. The weight loss above 400 °C was due to gradual decomposition of lignin.^25^ A 32% char yield was observed at 700 °C which is much higher than that of other bio‐derived carbons. For example, jute rope showed a yield of 11%, while kapok fibers produced carbon with a 20% yield.^18^, ^26^ This suggests that the carbonization of tea leaves is a high yield process and could be adopted for large scale production.

**Figure 2:**
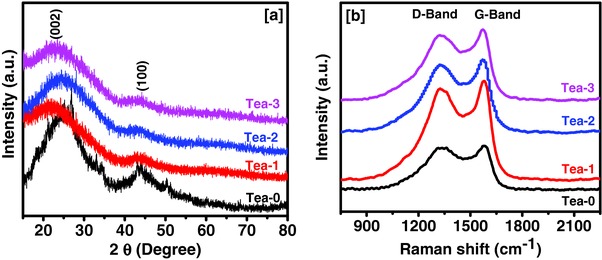
a) XRD patterns and b) Raman spectra of tea leaves derived carbons.

The structural and morphological characteristics of carbon from waste tea leaves were analyzed using X‐ray diffraction (XRD) with a Raman and scanning electron microscope. X‐ray diffraction patterns of unactivated and activated tea leaves are shown in the **Figure** **2**a. Two peaks around 2θ of 24° and 44° were observed for the activated tea leaves which corresponds to (002) and (100) planes of the graphitic carbon. It is interesting to note that peaks of the unactivated tea leaves have shifted toward a lower angle in activated carbon, indicating an increase in lattice parameters on KOH activation. Raman spectroscopy was used to determine graphitic and diamond phases of the carbon derived from tea leaves. Two characteristics peaks around 1340 and 1581 cm^−1^ were observed in all the samples, which can be assigned to D‐band and G‐band of carbon, respectively (Figure 2b). D‐band in carbon samples corresponds to the sp^3^ hybridized disordered carbon phase, while G‐band corresponds to sp^2^ hybridized graphitic phase of the carbon.^27^, ^28^ D‐band represents disorder in the carbon structure of the sample. The proportion of the disordered carbon presence in carbon samples can be described by the relative intensity of D‐band and G‐band (*I*_D_/*I*_G_ ratio). The *I*_D_/*I*_G_ ratio for unactivated tea leaves (Tea‐0) was 0.96 which decreased to 0.94, 0.93, and 0.92 for Tea‐1, Tea‐2, and Tea‐3 samples, respectively. The decrease in the *I*_D_/*I*_G_ ratio indicates that the graphitic phase increases with increasing amount of activating agent. It is worth noting that the *I*_G_/*I*_D_ ratio for all the tea leaves samples was more than 1 (e.g., 1.09 for Tea‐3 sample), which is higher than that of commercial activated carbon (*I*_G_/*I*_D_ = 0.52).^29^, ^30^ Commercial graphene nanoplates showed an *I*_G_/*I*_D_ value of 1.55.^30^ *I*_G_/*I*_D_ values of carbon derived from the tea leaves were higher than carbon derived from sugarcane bagasse and hemp.^29^, ^30^ Results indicate that higher concentration of graphitic phase, which is the conducting phase of the carbon, could be beneficial for electrochemical charge‐transfer by reducing series resistance and thus better energy storage performance can be expected.

**Figure 3:**
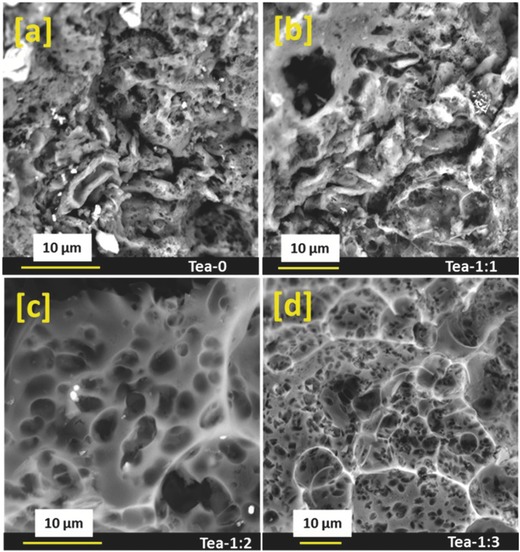
SEM image of unactivated (Tea‐0) and KOH activated (Tea‐1, Tea‐2, and Tea‐3) tea leaves.

Scanning electron microscope (SEM) images of unactivated and activated tea leaves derived carbon are shown in **Figure** **3**. It is clear from the SEM images that KOH activation modifies the surface of the tea leaves. The thermal treatment of unactivated tea leaves along with activating agent introduces porosity in tea leaves derived carbon. High porosity in the tea leaves derived carbon will provide higher surface area to the electrolyte ions and thus will improve charge storage capacity.^31^, ^32^ The introduced porosity using KOH as activation agent can be understood by the following expression

(1) $$6\mathrm{KOH}+2C\to 2K+3H_{2}+2K_{2}{\mathrm{CO}}_{3}$$

KOH reacts with the carbon in the tea leaves and forms the product shown in Equation (1). The formed products are decomposed and washed away after treating with 1 m HCl and water. The KOH reacted carbon in this process creates porous structure.^33^, ^34^ The porosity of the carbon can be controlled by varying the concentration of the activating agent.

**Figure 4:**
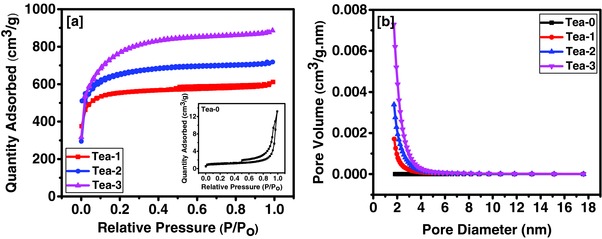
a) Nitrogen adsorption–desorption isotherms and b) BJH pore size distributions of unactivated and KOH activated tea leaves.

The effect of KOH activation on the surface area and porosity of the tea leaves was also studied using nitrogen adsorption–desorption isotherms of unactivated and activated tea leaves and results are displayed in **Figure** **4**a. It is evident from the nitrogen adsorption–desorption isotherms that the surface area and porosity of the tea leaves are highly dependent on the amount of activating agent used. Unactivated tea leaves (Tea‐0) showed very low N_2_ adsorption indicating almost nonexistence of the porous structure (inset of Figure 4a) whereas 1:1 KOH activated tea leaves (Tea‐1) displayed a combination of type I and IV isotherms where sharp slope at low pressure followed by steady increment in the N_2_ adsorption and the appearance of hysteresis curve over relative pressure of 0.5 was observed. This type of isotherm suggests presence of both micropores and mesopores structure in the carbon.^35^ On the other hand, 1:2 and 1:3 (tea leaves: KOH) activated tea leaves showed type I isotherm where most of the nitrogen absorption occurred at relative low pressure followed by almost constant absorption at higher relative pressures. Such behavior is characteristic of a material with a microporous structure. Brunauer–Emmett–Teller (BET) isotherm plot confirms the microporous nature of the tea leaves derived carbon. These observations were further confirmed by pore size distribution (PSD) from Barrett–Joyner–Halenda (BJH) plots (Figure 4b). The PSD plot for the tea leaves derived carbon showed a bimodal distribution of micropores and mesopores with maximum pores of the diameter less than 2 nm. Some of the important characteristics obtained via nitrogen adsorption–desorption isotherms are given in **Table** **1**. Unactivated tea leaves showed a surface area of 3.55 m^2^ g^−1^ which increased to 1780, 2090, and 2532 m^2^ g^−1^ for Tea‐1, Tea‐2, and Tea‐3, respectively, after activation. While, surface area of the tea leaves was observed to increase with increasing amount of KOH, the pore diameter first increased and then decreased with increasing KOH concentration. Based on the obtained results, it can be stated that a higher amount of activating agent etches the carbon deeper and creates higher surface area with higher pore volume.

**Table 1:** BET surface area, pore volume (*V*_total_), and pore diameters of unactivated and KOH activated tea leaves

Sample	BET surface area [m^2^ g^−1^]	*V*_total_ [cm^3^ g^−1^]	Pore diameter [nm]
Tea‐0	3.55	0.001	–
Tea‐1	1780	0.279	0.627
Tea‐2	2090	0.646	1.236
Tea‐3	2532	0.681	1.075

**Figure 5:**
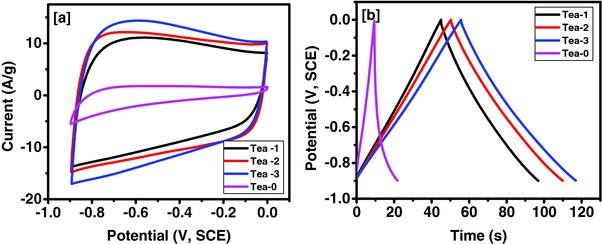
a) CV curves at 100 mV s^−1^ and b) potential–time curves at 2 A g^−1^ for unactivated and KOH activated tea leaves in 3 m KOH electrolyte.

Electrochemical characterizations of the unactivated and KOH activated tea leaves were performed to understand the effect of surface area and porosity on their charge storage capacity. **Figure** **5**a compares the CV curves of unactivated and activated tea leaves at 100 mV s^−1^ in 3 m KOH electrolyte in potential range of −0.9 to 0 V (vs SCE). Two important phenomena were observed, first, the area under the CV curves (and thus the charge storage capacity) was maximum for Tea‐3 sample while, it was least for Tea‐0 having distorted rectangular CV curve. This indicates low charge storage capacity of Tea‐0 compared to activated Tea‐3 sample. Second, the CV curves were almost rectangular in shape without any redox peaks, indicating near to ideal behavior of electrochemical double layer type capacitor.^16^, ^36^ Furthermore, the charge–discharge characteristics of the electrode based on tea leaves were compared at current density of 2 A g^−1^ and are shown in Figure 5b. The shortest and the largest discharge time were observed for Tea‐0 and the Tea‐3 sample, respectively, suggesting the highest charge storage capacity was the Tea‐3 sample. As seen in both CV and GCD curves, the chemical activation had a significant effect on the charge storage capacity of the tea leaves. Based on these results, Tea‐3 sample was carried forward for detailed electrochemical characterizations for its potential application in supercapacitor devices.

**Figure 6:**
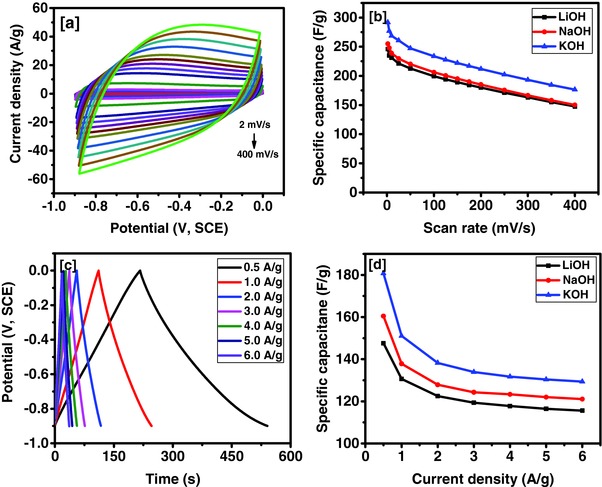
a) CV curves in 3 m KOH electrolyte, b) specific capacitance versus scan rate plots in 3 m KOH, NaOH and LiOH electrolytes, c) potential–time curves at various current densities in 3 m KOH electrolyte, and d) specific capacitance versus current density plots for Tea‐3 in 3 m KOH, NaOH, and LiOH electrolytes.

The effect of scan rate and size of electrolyte ions on the charge storage capacity of the Tea‐3 sample was studied. **Figure** **6**a shows the CV curves of the Tea‐3 sample at various scan rates in KOH electrolyte. The electrochemical double layer mechanism was confirmed by the observation of the nearly rectangular shape of the CV curves and absence of any redox peak. Electrolyte ions undergo reversible absorption and desorption reactions over the porous surface of the activated tea leaves. The shape of the CV curves was retained even at higher scan rates, indicating a fast charge‐transfer process from the electrochemical double layer. Specific capacitance (*C*) from the CV data was calculated using the following expression

(2) $$C\left(\mathrm{F/g}\right)=\frac{A}{\Delta V\times \left(\;\frac{\partial v}{\partial t}\right)\times m}$$

where *A* is the area under the CV curve, $\frac{\partial v}{\partial t}$ is the scan rate, Δ*V* is the potential window, and *m* is the mass of the Tea‐3 sample. Figure 6b, shows the variation of specific capacitance as a function of scan rates and electrolytes. Higher specific capacitance was observed at a lower scan rate in all the studied electrolytes. Low specific capacitance at higher scan rates is due to lack of time for electrolyte ions to absorb and desorb on the surface of the electrode. The maximum specific capacitance of 292 F g^−1^ was observed in 3 m KOH electrolyte, while the lowest specific capacitance of 246 F g^−1^ was obtained in 3 m LiOH electrolyte at scan rate of 2 mV s^−1^. **Table** **2** shows the comparison between activated tea carbon and other reported bio‐based derived carbon. From the overall observation, it can be concluded that activated tea could be one of the most efficient materials used for supercapacitor electrodes.

**Table 2:** Comparison of the tea leaves derived carbon to carbon derived from other biomass

Name	Activating agent	Surface area [m^2^ g^−1^]	Highest specific capacitance [F g^−1^]	Reference
Argan seed shell	KOH and (NH_4_)_2_S_2_O_8_	1654	228	43
Palm‐empty fruit bunch	KOH + CO_2_	1704	149	16
Tri‐doped carbon	Microwave with ammonium polyphosphate	228	170	44
Corn straw	KOH	894	166	36
Corn straw‐nitrogen doped	KOH	1252	181	36
Cellulose‐based activated nanofibers (ACNF)	Steam	865	130	45
Multiwall carbon nanotubes (MWCNT)/ACNF	Steam	1120	160	45
Bamboo @MnO_2_	HCl	–	174	46
Banana peel	Hydrothermal	1358	126	47
N‐doped banana peel CF	Hydrothermal	648	186	47
Activated waste tire	KOH	1625	135	48
Coffee ground	Microwave with ammonium polyphosphate	1000	286	49
Coffee shell	ZnCl_2_	842	156	9
Oil palm kernel shell	KOH	462	210	50
Oil palm kernel shell‐P doped	Steam	727	123	50
Waste paper	KOH	416	180	20
Coconut shell	Steam + pyrolysis	1532	228	25
Rice husk	CO_2_	1357	106	5
Tea (1:3)	KOH	2532	292	This work

The galvanostatic charge–discharge measurements were used to further study the electrochemical behavior of the Tea‐3 sample. Figure 6c shows the charge–discharge characteristics of the Tea‐3 sample in KOH electrolyte. The linear potential curve over time along with symmetry suggested nearly ideal behavior of the electrode material. As the value for current density decreased, the time for galvanostatic charge discharge increased. The discharge time is directly related to the specific capacitance (*C*) of the electrode through the following equation

(3) $$C\left(\mathrm{F/g}\right)=\frac{I\times \Delta t}{\Delta V\times m}$$

where *I* is the discharge current (A), Δ*t* is the discharge time (s), Δ*V* is the potential window (V), and *m* is the mass (g) of the tea leaves. Change in specific capacitance as a function of current densities is shown in Figure 6d. Specific capacitance was observed to decrease with increasing current densities, which could be due to lack of time available for the electrolyte ions to disseminate within the pores of the tea leaves. The highest specific capacitance of 181 F g^−1^ was observed at a current density of 0.5 A g^−1^ in KOH electrolyte. Based on this observation, KOH has proven to be a better electrolyte than NaOH and LiOH electrolytes. The highest specific capacitance for the Tea‐3 in KOH electrolyte could be associated with the smallest hydrated ionic radius of K^+^ (3.31 Å). The hydrated ionic radius of the Na^+^ (3.58 Å) and Li^+^ (3.82 Å) are bigger than that of K^+^. Since the ionic conductivity of the ions decreases with increasing size, K^+^ ions provide the highest ionic conductivity and thus can get access to most of the pores within same time frame which results in higher charge storage capacity.^37^

**Figure 7:**
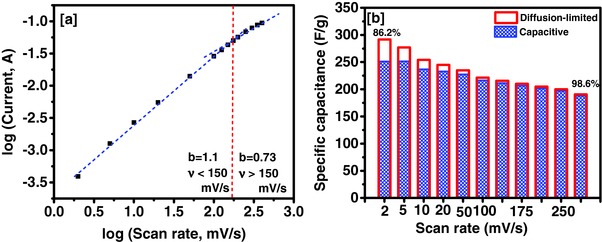
a) Plot of log I versus log v for Tea‐3 sample to determine the value of b, and b) variation of specific capacitance as a function of scan rate showing capacitive and diffusion‐limited contribution to total capacitance.

The current observed in the CV curves can be described using the power law behavior as given below^38^

(4) $$i=av^{b}$$

where *i* is the cathodic current (A) at −0.45 V, *v* is the scan rate (mV s^−1^), and *a* and *b* are the variables. The value of *b* defines the charge storage mechanism. A capacitive mechanism is the main charge storage mechanism if *b* equals to 1. The main charge storage mechanism is considered as diffusion limited if *b* equals to 0.5. The value of *b* for Tea‐3 was observed to be 1.1 and 0.73 at low scan and high scan rates, respectively, as shown in **Figure** **7**a. The capacitive and diffusion‐limited contributions to overall contribution can be determined using the following expression^39^

(5) $$i=k_{1}v+k_{2}v^{1/2}$$

where *k*_1_ and *k*_2_ are suitable values. The *k*_1_ *v* is the contribution due to capacitive part while *k*_2_ *v* ^1/2^ is the diffusion‐limited contributions to overall observed current. The value of *k*_1_ and *k*_2_ was determined by plotting *i*/*v* ^1/2^ versus *v* ^1/2^. The capacitive and diffusion‐limited contribution to overall capacitance for the Tea‐3 is shown in Figure 7b. As seen, the capacitive contribution increases with increasing scan rates. Tea sample showed 86.2% of capacitive contribution at 2 mV s^−1^ which increased to 98.6% at 300 mV s^−1^.

**Figure 8:**
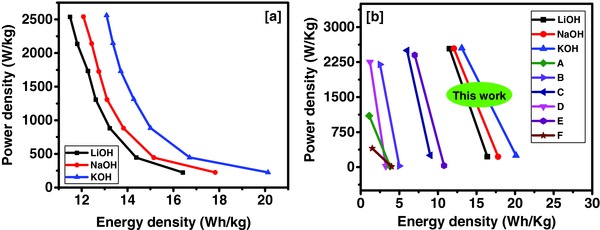
Ragone plots for: a) Tea‐3 sample for different electrolytes, b) Comparison with other results (A—commercial activated carbon,^40^ B—commercial mesoporous activated carbon,^41^ C—sugarcane activated carbon,^29^ D—bamboo activated carbon,^12^ E—phosphorus doped carbon,^41^ F—sago bark activated carbon^42^).

**Figure 9:**
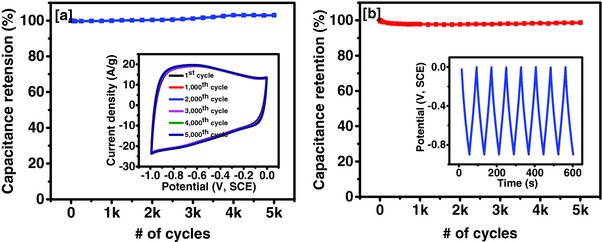
a) Capacitance retention verse number of CV scans, inset figure shows CV curves at various cycles, and b) capacitance retention verse number of charge–discharge cycles, inset figure shows potential verses time plot for first few cycles in 3 m KOH.

Energy density (*E*) and power density (*P*) of the Tea‐3 were calculated using the expressions given below

(6) $$E(\mathrm{Wh/kg})=\frac{C\times \;\Delta V^{2}}{7.2}$$

(7) $$P(\mathrm{W/kg})=\frac{E\times 3600}{t}$$

where *C* is the specific capacitance of the electrode determined using charge–discharge measurements, Δ*V* is the potential window (V), and *t* is the discharge time (s). The relation between energy and power densities is shown in Ragone plots for Tea‐3 in all three studied electrolytes (**Figure** **8**a). The highest energy density of 20 Wh kg^−1^ and power density of 2.56 kW kg^−1^ were observed in 3 m KOH electrolyte, which is comparatively higher to most of the activated carbon samples from previous reports (Figure 8b). The long‐term performance of the Tea‐3 was studied using cyclic voltammetry and galvanostatic charge–discharge measurements for 5000 cycles (**Figure** **9**). As seen in the capacitance versus number of cycle plots, the electrode showed a very stable performance in CV and CD‐based long‐term stability test.

**Figure 10:**
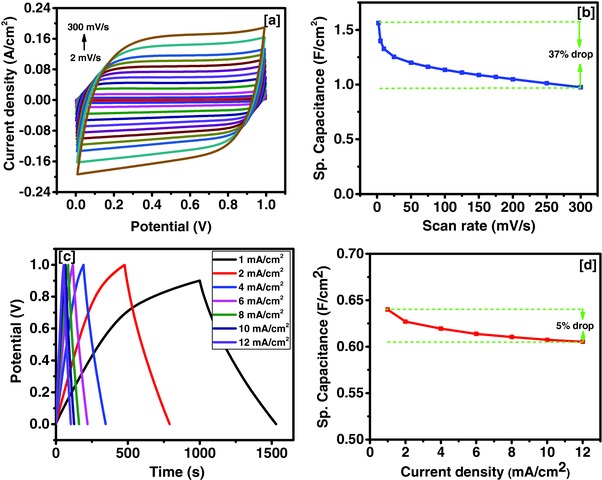
a) CV curves at various scan rates, b) variation of specific capacitance versus scan rates, c) charge–discharge characteristics, and d) variation of specific capacitance versus applied current for supercapacitor device in 3 m KOH electrolyte.

**Figure 11:**
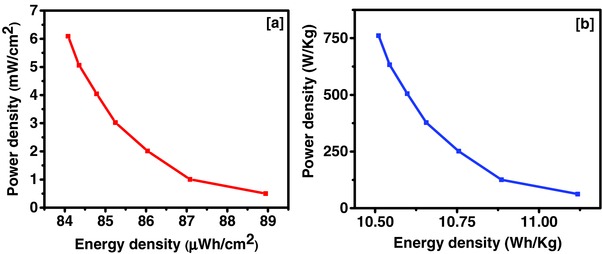
Ragone plot for the symmetrical supercapacitor device using Tea‐3 sample a) areal and b) gravimetric energy and power densities.

The potential application of tea leaves as energy storage devices was investigated by fabricating a symmetrical supercapacitor device. The electrochemical performance of the device was studied in 3 m KOH using CV, GCD, and EIS. The CV curves of the device at various scan rates are shown in **Figure** **10**a. The CV curves were almost identical in shape and rectangular even at higher scan rates indicating high rate stability of the device. The device showed the highest specific capacitance of 1.56 F cm^−2^ at 2 mV s^−1^ and about 63% of the specific capacitance was retained at high scan rate of 300 mV s^−1^ (Figure 10b). As mentioned above, the observed current in the CV measurements follow the power law behavior as given in Equation (1). The value of *b* was calculated to be 0.95, indicating charge storage mechanism in tea leaves‐based device is mainly capacitive in nature. The charge storage capacity of the device was further tested using galvanostatic charge–discharge measurements. The potential–time curves for the device at various current densities are shown in Figure 10c. Variation of specific capacitance as a function of current density showed a maximum specific capacitance of 0.64 F cm^−2^ at 1 mA cm^−2^ (Figure 10d). About 95% of the charge storage capacity was retained even after increasing current density from 1 to 12 mA cm^−2^, confirming high rate stability of the device. The Ragone plot for the symmetrical supercapacitor device is given in **Figure** **11**. In **Table** **3**, areal energy and powder density for various other materials are compared. It can be clearly seen that energy and power densities for the tea leaves derived carbon are among the best‐reported values. Observed high energy and power density could enable its potential use for fast‐charge supercapacitor batteries. The gravimetric energy and power densities of the tea‐based symmetrical supercapacitor device was compared with other reports in **Table** **4**. The gravimetric energy density of tea‐based device was comparable with other devices. High gravimetric energy density could be beneficial for realizing supercapacitor batteries where high energy density is one of the important parameters.

**Table 3:** Comparison of areal energy and power densities with other reports

Device	Energy density [μWh cm^−2^]	Power density [mW cm^−2^]	Reference
Multiwall nanotubes (MWNT)/Carbon nanofibers (CNF)	9.8	8.07	51
Polypyrrole (PPy)/Graphene oxide (GO)	12.9	5.8	52
GO/PPy (aqueous)	16.8	4	53
Graphene‐cellulose paper	≈6	≈17	54
Carbon nanotubes (CNT)	≈33	≈14	55
Reduced GO (rGO)‐Polyaniline‐nanofibers (PANI‐NFs)‐3D	≈20	≈30	56
rGO‐Poly(3,4‐ethylenedioxythiophene)‐poly(styrenesulfonate) (PEDOT/PSS)	34	≈98	57
Tea‐3	89	6.1	This work

**Table 4:** Comparison of gravimetric energy and power densities with other reports

Device	Energy density [Wh kg^−1^]	Power density [W kg^−1^]	Reference
Catalyst‐free carbon nanospheres	5	400	42
Activated carbon (AC)	≈6.5	4000	36
N‐doped AC	≈7.5	4000	36
3D graphene/MnO_2_	6.8	2500	58
Graphene/graphene composite	2.8	≈2500	59
MnO_2_ nanowire/graphene composite	5.2	≈2500	59
3D graphene oxide‐polypyrrole	16.4	4000	53
3D graphene oxide‐polyaniline	≈12	≈10 000	56
Tea‐3	11.1	761	This work

**Figure 12:**
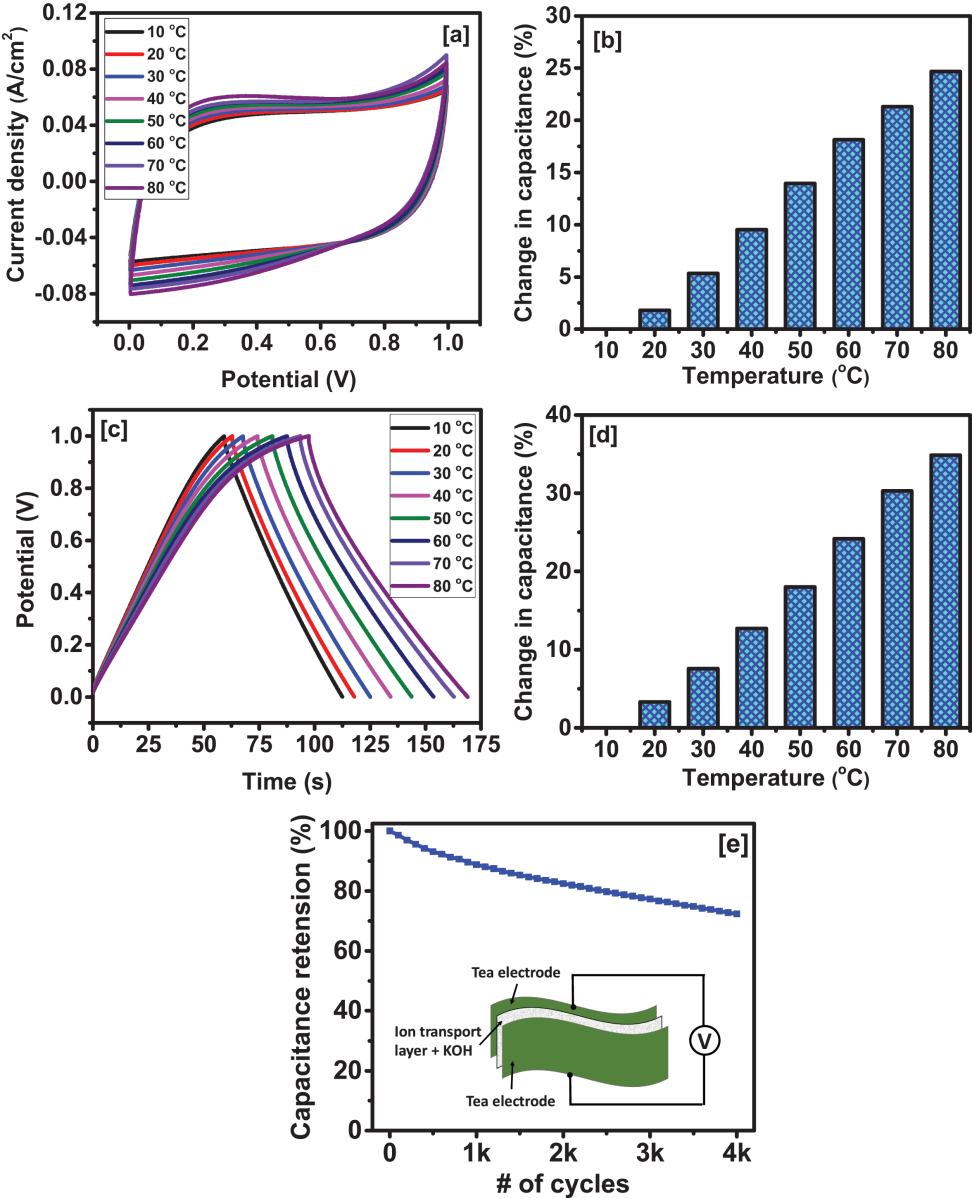
a) CV curves of the device at various temperatures at 100 mV s^−1^, b) change in capacitance versus temperature plots using CV data, c) galvanostatic charge–discharge behavior of the device at various temperatures (measured at 10 mA cm^−2^), d) change in capacitance versus temperature of the device measured at different current densities in 3 m KOH electrolyte, and e) capacitance retention verse number of cycles for the device at 70 °C, inset figure shows schematic of the device.

The effect of temperature on the electrochemical properties of the symmetrical supercapacitor device was studied in detail. The CV curves of the device at various temperatures showed identical and rectangular shape similar to ideal capacitors (**Figure** **12**a). The area under the CV curves was observed to increase with increasing temperature without distorting the shape, indicating improvement in charge storage capacity and high temperature stability of the activated tea leaves derived carbon. A 25% improvement in the charge storage capacity was observed when temperature was raised from 10 to 80 °C (Figure 12b). Additional study on effect of temperature on electrochemical properties of the device was studied using galvanostatic charge–discharge method. Discharge time increased with temperature confirming the improvement in charge storage capacity (Figure 12c). Charge storage capacity was improved about 35% on increasing temperature from 10 to 80 °C (Figure 12d). Similar behavior was observed in our past results as well as other reports where, with increasing temperature the charge storage capacity of the electrode material increased.^60^, ^61^ The cyclic stability of the device was further studied at elevated temperature using galvanostatic measurement. The cyclic performance of the device at 70 °C is shown in Figure 12e. About 72% of the initial charge storage capacity was maintained after 4000 cycles, suggesting good cyclic stability at high temperature.

**Figure 13:**
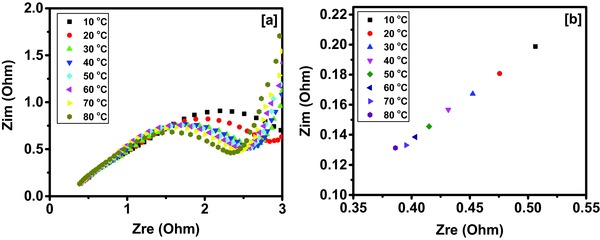
a) Nyquist plots of the device at various temperatures and b) zoomed Nyquist plots of the device at various temperatures.

Electrochemical impedance spectroscopy was used to understand the effect of temperature on the charge storage capacity of the device. The Nyquist plots at various temperatures of the Tea‐3‐based supercapacitor device are given in **Figure** **13**a. As seen, the arch of the semicircle decreases with increase in temperature indicating reduction in charge transfer resistance of the device. The enlarged section of Nyquist plots (Figure 13b) shows that the equivalent series resistance (ESR) decreases with increasing temperature, which could be due to increased mobility of the ions in the electrolyte. This suggests that the improved performance of the device at higher temperatures could be due to depreciation of charge transfer resistance and ESR of the device with respect to temperature.

## 3 Conclusions

Highly porous carbon derived from tea leaves was prepared via a cost effective chemical activation method using KOH as activating agent. The higher mass ratio of KOH generated carbon with a larger surface area and higher concentration of graphitic phase. The activated tea leaves showed a maximum specific capacitance of 292 F g^−1^ at 2 mV s^−1^ in 3 m KOH with outstanding cyclic stability (≈100%) up to 5000th cycle of study. A supercapacitor device based on high surface area carbon from tea leaves showed high rate stability with only 5% of the capacitance lost by increasing current density from 1 to 12 mA cm^−2^. The device displayed high energy and power densities with improved charge storage capacity at elevated temperature. Our work suggests that used tea leaves can be utilized for fabrication of eco‐friendly, high performance, and durable supercapacitors which could operate at elevated temperatures.

## 4 Experimental Section

*Preparation of Activated Bio Porous Tea Carbon*: All chemicals used in this work were analytical grade and used as received. Used household tea was thoroughly washed, cleaned, and dried at 60 °C in an oven. These dried tea leaves were ground into a very fine size for calcination and KOH activation. Carbonization was carried out directly without any pretreatment for 2 h at 450 °C under inert nitrogen. Furthermore, resultant carbon powder was chemically activated using KOH. For chemical activation, 1 g of obtained powdered tea leaves and 1 g of KOH pellets (1:1 wt. ratio) were thoroughly mixed and pyrolyzed at 800 °C (5 °C min^−1^) for 2 h under nitrogen atmosphere. The collected black powder was ground, washed with 1 m HCl solution and deionized water. The activated tea leaves were dried at 60 °C for overnight. The obtained activated carbon from tea leaves was named as Tea‐1. Similarly, carbons obtained by activation of tea leaves with different mass ratios of KOH (1:2 and 1:3) were named Tea‐2 and Tea‐3. The tea leaves without KOH activation were named Tea‐0.

*Characterization*: X‐ray powder diffraction (Shimadzu X‐ray diffractometer) was used to determine the crystallite structure of the carbon that was prepared from tea leaves using 2*θ–θ* scan with Cu*K*_α1_ (λ = 1.5406 Å) radiation. Argon ion laser Raman spectroscopy was used to determine G and D bands of carbon using a wavelength of 514.5 nm as the excitation source (Model Innova 70, Coherent). Microporous structure and morphology of all the tea samples were determined using field emission scanning electron microscopy (JEOL 7000 FE‐SEM). Nitrogen adsorption/desorption isotherms were analyzed after degassing the samples for 24 h at 90 °C using a Micrometrics, USA, ASAP 2020 volumetric adsorption analyzer at 77 K. BET absorption method was used to determine specific surface area while, pore size distributions were calculated using BJH theory. Thermogravimetric analysis (TGA) of samples was performed using a TA instrument (TA 2980) to determine weight loss with respect to temperature under nitrogen atmosphere at a rate of 10 °C min^−1^.

*Electrochemical Measurements*: Electrochemical characterizations of the unactivated and activated carbon derived from tea leaves were evaluated using a three‐electrode system. Working electrodes were prepared by coating the viscous mixture of carbon from tea leaves (80 wt%), acetylene black (10 wt%), and polyvinylidene difluoride (10 wt%) in *N*‐methyl pyrrolidinone on to a nickel foam. A three‐electrode system comprised of tea sample coated nickel foam as a working electrode, a platinum strip as a counter electrode, and saturated calomel electrode (SCE) as a reference electrode were used. Three different electrolytes (3 m KOH, NaOH, and LiOH) were used for the electrochemical measurements. A symmetrical supercapacitor was fabricated by sandwiching an ion transporting layer between two identical tea leaves‐based electrodes in 3M KOH electrolytes. The effective device size was 0.25 cm^2^ with 8 mg of total weight of the active material. A Versa STAT 4‐500 electrochemical workstation (Princeton Applied Research, USA) was used to perform cyclic voltammetry, galvanostatic charge–discharge, and electrochemical impedance spectroscopy. Electrochemical impedance spectra were recorded in the frequency range of 0.05 Hz to 10 kHz with 10 mV of AC voltage amplitude. The electrochemical performance of the device was also studied at elevated temperatures. For these measurements, the device was placed in a glass cell with a water jacket and the glass cell was filled with 3M KOH electrolyte. The temperature of the electrolyte and device was controlled using Fisher Scientific isotemp refrigerated/heated bath circulators (AC 25).

## Conflict of Interest

The authors declare no conflict of interest.
